# Risk Factors for Bloodstream Infections Due to ESBL-Producing *Escherichia coli*, *Klebsiella* spp., and *Proteus mirabilis*

**DOI:** 10.3390/pharmacy11020074

**Published:** 2023-04-13

**Authors:** Mary Kathryn Vance, David A. Cretella, Lori M. Ward, Prakhar Vijayvargiya, Zerelda Esquer Garrigos, Mary Joyce B. Wingler

**Affiliations:** 1 Department of Pharmacy, Vanderbilt University Medical Center, Nashville, TN 37232, USA; maryk.vance@vumc.org; 2 Department of Antimicrobial Stewardship, University of Mississippi Medical Center, Jackson, MS 39216, USA; dcretella@umc.edu; 3 Division of Infectious Diseases, Department of Medicine, University of Mississippi Medical Center, Jackson, MS 39216, USA; pvijayvargiya@umc.edu (P.V.); zesquergarrigos@umc.edu (Z.E.G.); 4 Department of Population Health Science, University of Mississippi Medical Center, Jackson, MS 39216, USA; lward@umc.edu

**Keywords:** antibiotics, bloodstream infection, extended-spectrum beta lactamase

## Abstract

(1) Background: Risk factors for extended-spectrum beta-lactamase (ESBL) infections could vary geographically. The purpose of this study was to identify local risk factors for ESBL production in patients with Gram-negative bacteremia. (2) Methods: This retrospective observational study included adult patients admitted from January 2019 to July 2021 and had positive blood cultures for *E. coli*, *K. pneumoniae*, *K. oxytoca*, and *P. mirabilis*. Patients with ESBL infection were matched to a non-ESBL-producing infection with the same organism. (3) Results: A total of 150 patients were included: 50 in the ESBL group and 100 in the non-ESBL group. Patients in the ESBL group had a longer length of stay (11 vs. 7 days, *p* < 0.001), but not increased mortality (14% vs. 15%, *p* = 0.87) Multivariate analysis identified the receipt of >1 antibiotic in the last 90 days as a risk factor for ESBL infection (OR = 3.448, 95% CI = 1.494–7.957; *p* = 0.004); (4) Conclusions: Recent antimicrobial use was identified as an independent risk factors for ESBL-producing Enterobacterales infections. Knowledge of this risk may improve empirical therapy and reduce inappropriate use.

## 1. Introduction

Extended-spectrum beta-lactamases (ESBLs) are a group of enzymes that confer resistance to most beta-lactam antibiotics, including broad-spectrum cephalosporins [1]. Many Gram-negative bacteria are capable of producing ESBL, but they are most commonly seen in *Klebsiella pneumoniae*, *Klebsiella oxytoca*, and *Escherichia coli* [2]. There are several different varieties of ESBL, including TEM, SHV, CTX-M, and OXA, and each has variable levels of activity against particular beta-lactam substrates. Beta-lactamase production differs on the basis of geographic location. In the United States, the highest overall ESBL production rates was for the mid-Atlantic and west–south central regions, with CTX-M and SHV as the most common varieties. CTX-M was more prevalent in West South Central, mid-Atlantic, and East North Central regions, while SHV was more prevalent in the South Atlantic, mid-Atlantic, and East South Central regions [3]. 

In the United States, the incidence of infection with ESBL-producing Enterobacterales (ESBL-E) appears to be increasing, creating a significant problem for empirical antibiotic prescription [4]. A study performed in the United States evaluated the change in the rates of ESBL-producing *E. coli* in urine samples between 2014 and 2020 [5]. The overall rates of ESBL increased from 6% (2014) to 10% (2020). When stratified by the type of infection, the rate of healthcare-onset/associated ESBL *E. coli* urine cultures rose much more rapidly, at 2.31% per year. Community-onset infections also increased at the rate of 0.91% per year. This study does not include information about ESBL rates during the COVID-19 pandemic, and recent data suggest that an even greater rise in ESBL infections has occurred since 2020 [6]. In 2022, the Centers for Disease Control and Prevention (CDC) published a special report: COVID-19: U.S. Impact on Antimicrobial Resistance [6]. ESBL-E infections are categorized as a “serious threat” by the CDC and continued to be tracked during the pandemic. That report compared the rates of multidrug-resistant organisms (MDROs) in 2019 and 2020, and found that the rate of ESBL-E infections increased dramatically, by 10% overall (7% for community-onset, and 32% in hospital-onset infections). Inappropriate antibiotic use during the pandemic may have contributed to this rise in ESBL-E and other MDRO infections. On the basis of CDC data, around 80% of hospitalized patients with COVID-19 had received an antibiotic between March and October 2020 [6]. 

Infections caused by ESBL-producing organisms are associated with higher morbidity and mortality compared to those caused by more susceptible organisms, which may be driven by inappropriate empirical therapy [7]. A retrospective cohort study of 354 patients with community-acquired *E. coli* bacteremia found that mortality was 60.8% in patients with infections caused by ESBL-producing organisms, compared to 23.7% in patients with infections caused by non-ESBL producing organisms [8]. 

Treating patients with ESBL infections can be challenging due to the limited treatment options [1]. ESBL-encoding plasmids frequently carry resistance genes for other, non-beta-lactam antimicrobial classes, including fluoroquinolones, aminoglycosides, and sulfonamides, limiting beta-lactam alternatives [9]. Among beta-lactams, carbapenems are effective against ESBLs and generally recommended for ESBL bacteremias, but indiscriminate use could lead to increased rates of carbapenem resistance [10]. Furthermore, carbapenem use was identified as a risk factor for carbapenem-resistant Enterobacterales acquisition [11]. Therefore, identifying patients at risk for ESBL infections could assist in determining patients for whom empirical carbapenem therapy would be appropriate. Risk factors for ESBL include malignancy, urinary catheterization, and urinary-tract infections, recent antibiotic treatment, diabetes mellitus, a history of ESBL infections, stomach-tube catheterization, central lines, mechanical ventilation, a longer length of hospital stay, and recent outpatient procedures [12,13,14,15,16]. Since risk factors vary significantly by location, previously published studies recommend determining institution-specific risk factors for ESBLs. The rate of ESBL production among these organisms at our institution is approximately 11%, rendering early identification critical for appropriate empiric therapy. Therefore, the objective of this study is to identify risk factors for ESBL production in patients at our hospital with bacteremia due to Enterobacterales. 

## 2. Materials and Methods

### 2.1. Study Design

A retrospective cohort study was conducted on adult patients admitted to a large academic medical center in the East South Central United States. This study was approved by the University of Mississippi’s Medical Center Institutional Review Board (protocol number 2021-1052). A computerized database was used to identify patients with blood cultures that grew *Escherichia coli*, *Klebsiella pneumonia*, *Klebsiella oxytoca*, and *Proteus mirabilis* from 1 January 2019 to 31 July 2021. Patients were divided into two groups on the basis of the presence of ESBL. ESBL-production was defined as the nonsusceptibility to ceftriaxone and/or ceftazidime, in addition to a positive phenotypical test (Vitek^®^ 2; Biomérieux, Marcy l’Etoile, France). Patients with infection due to a non-ESBL-producing isolate were matched to those with an infection due to an ESBL-producing isolate of the same organism in a 2:1 ratio, and a convenience sample was selected for analysis. In patients with more than one eligible culture during the study period, only data from the first episode of bacteremia were included. Prisoners, pregnant patients, patients with plasmid-mediated AmpC or carbapenem-resistant isolates, and those with polymicrobial cultures were excluded. Clinical data were collected through a review of electronic medical records: patient demographics, clinical characteristics, microbiological data, chronic medical conditions, laboratory data, current and past antibiotic regimens, and other relevant information. Data were collected on password-protected database REDCap [17]. 

### 2.2. Study Objectives 

The primary aim of this study was to identify risk factors for ESBL production in patients with Gram-negative bacteremia due to *E. coli*, *K. pneumonia*, *K. oxytoca*, and *P. mirabilis*. Secondary aims were to compare the inpatient mortality rates between patients with ESBL and non-ESBL infections, evaluate if ESBL production affected the length of stay, and determine the time to the initiation of treatment with a carbapenem in patients with ESBL infections.

### 2.3. Definitions

A nosocomial infection was defined as a positive blood culture that had occurred later than 48 h after admission to the hospital. A healthcare-associated infection was defined as positive blood culture obtained within 48 h of admission from patients who had received intravenous (IV) therapy or specialized nursing care at home, attended a hospital or hemodialysis clinic, received IV chemotherapy in the past 30 days, or resided in a long-term care facility prior to admission. A community-acquired infection was defined as a positive blood culture within the first 48 h of hospitalization in patients who did not fit the criteria for healthcare-associated infection. Immunosuppressive therapies included corticosteroid therapy for at least 2 weeks or cancer chemotherapy, radiation therapy, immunological therapies such as adalimumab or etanercept, and transplant medications such as tacrolimus and cyclosporine within 30 days prior. Urological procedures included transurethral prostate or bladder surgery, ureteroscopy including lithotripsy, percutaneous stone surgery, and urological stent placement prior to admission. The potential source of bacteremia was determined according to the identification of the same organism in a nonblood culture. If no positive culture results from alternative sites were available, the potential source of infection was determined on the basis of progress notes in the patient’s medical record. If no potential source was mentioned, the bacteremia was classified as of unknown origin. Prior infections or colonization with ESBL-producing organisms referred to their documented growth in any clinical culture site in both inpatient and ambulatory settings within 12 months prior. Antibiotic administration within 90 days was considered the administration of at least one dose of any of these antibacterial agents. Days of therapy included any day that a patient had received at least one antimicrobial dose. 

### 2.4. Statistical Analysis

Descriptive and inferential statistical tests were performed to analyze data. Categorical data were analyzed using the two-tailed chi-squared or Fisher’s exact test, as appropriate Continuous variables were analyzed using the Student’s *t*- or Mann–Whitney U test as appropriate. Logistic regression analysis was used for the univariable and multivariable calculation of risk factors and odds ratios with 95% confidence intervals. Multivariate analysis was limited to five variables, including all significant variables from the univariate analysis. Statistical analysis was performed with SPSS 27 (IBM, 2019) and a *p*-value of <0.05 was statistically significant. 

## 3. Results

A total of 493 patient charts were screened for enrollment. Of these, 150 patients were included: 100 in the non-ESBL group, and 50 in the ESBL group. The most common reason for exclusion was polymicrobial cultures (24%), followed by cefoxitin resistance (8%), admission to a different hospital campus (7%), and pregnancy (4%). Baseline demographics were similar between the groups (Table 1). The urinary tract was the most common source of infection in both groups. The sources of infection were generally similar between the groups, but significantly more patients in the non-ESBL group had an intra-abdominal infection. 

Most patients empirically received antipseudomonal β-lactams, and the only significant difference was that a larger number of patients in the ESBL group had received meropenem compared with the non-ESBL group (22% vs. 2%, *p* < 0.001) (Table 2). Of the 11 patients who were empirically prescribed meropenem, 6 had a history of an ESBL infection. Both groups had a similar total duration of therapy. However, patients in the ESBL group had a longer duration of IV therapy (14 vs. 6 days, *p* < 0.001) and fewer transitions to oral therapy (50% vs. 4%, *p* < 0.001). 

Characteristics included in the univariate analysis are shown in Table 3. ESBL patients had had more antibiotic courses in the past 90 days (3 vs. 2, *p* < 0.001) and were more likely to have a history of ESBL infection (18% vs. 0%, *p* < 0.001). Patients in the ESBL group were more likely to have a healthcare-associated or nosocomial-acquired infection, while more patients in the non-ESBL group had a community-acquired infection. Patients in the ESBL group were also significantly more likely to have had a central line present and to be taking an immunosuppressive agent prior to admission.

Characteristics included in the multivariate analysis are shown in Table 4. The receipt of more than one antibiotic within the past 90 days was the only independently associated variable with ESBL bacteremia (OR = 3.448, 95% CI = 1.494–7.957; *p* = 0.004). 

For secondary outcomes, inpatient mortality was similar between the groups, namely, 15% in the non-ESBL group and 14% in the ESBL group (*p* = 0.87). The length of stay was significantly longer in the ESBL group (11 vs. 7 days; *p* < 0.001). The median time to the initiation of a carbapenem in the ESBL group was 2 days (IQR, 1–3 days). 

## 4. Discussion

In this retrospective study, the receipt of more than one antibiotic in the past 90 days and a history of ESBL infection were significant risk factors for bacteremia caused by ESBL-E. These findings are consistent with the results of previous studies [7,18]. Risk factors described by Augustine and colleagues included outpatient procedures within 30 days, prior infections or colonization with ESBL-E, and the number of prior courses of beta-lactams and/or fluoroquinolones used within the past 3 months [18]. Similarly, Freeman and colleagues identified previous colonization with ESBL-E and previous exposure to fluoroquinolones and first-generation cephalosporins as risk factors for ESBL-E bacteremia [7]. While the effect of different antibiotic classes was not directly assessed, beta-lactams and fluoroquinolones were the two most common antibiotic classes that patients in the ESBL group had received in the past 90 days. The risk of ESBL-E infection in patients with previous overall antibiotic exposure was similar, with previous studies finding odds ratios of around 3 to 4 [15,16]. 

Some previously identified risk factors failed to demonstrate a correlation in the study population. This may have been due to the low prevalence in the observed population. For example, undergoing a urological procedure in the past 90 days was identified as a risk factor [18,19]. There were only two patients included in this study who had undergone a urological procedure. This may also reflect our patient population and reinforces the need for institution-specific risk factors. Community-acquired infections are a negative predictor of ESBL production [15]. Only 10% of our ESBL group had a community-acquired infection. Despite the low rate of community-acquired ESBL-E BSI in our study, there are rising rates across the United States [20,21]. Local ESBL rates should be frequently monitored to ensure that adequate empiric treatment is initiated for patients with Enterobacterales infections. Pharmacists are in a good position to assist in monitoring local ESBL rates. On the basis of the CDC’s Core Elements of Hospital Antibiotic Stewardship Programs, all programs should have a pharmacist involved, and these individuals are tasked with reporting local resistance rates to prescribers, nurses, and hospital leaders [22]. 

Carbapenems were historically considered the agents of choice for ESBL-E infections. Prior to the availability of prospective data, results were mixed on whether carbapenem alternatives could be used for serious ESBL infections such as bacteremias. Several studies demonstrated no difference in clinical outcomes for beta-lactam/beta-lactamase inhibitors (BL/BLI) compared with carbapenems [23]. For example. Rodriguez-Bano and colleagues performed post hoc analysis of six prospective cohorts and found no difference in mortality at 30 days in patients with ESBL *E. coli* treated with BL/BLI or carbapenem [23]. On the other hand, Tamma and colleagues performed a similar comparison of piperacillin/tazobactam and carbapenems for ESBL bacteremias, and results demonstrated a 1.92 times higher risk of death in the piperacillin/tazobactam group (95% CI 1.07–3.45; *p* = 0.03) [24]. The MERINO trial supported the findings of the latter study and provided the first prospective clinical trial evidence supporting carbapenems as the antibiotics of choice in ESBL bacteremias [25]. That trial randomized 379 patients to either meropenem and piperacillin/tazobactam for the treatment of ESBL *E. coli* and *K. pneumoniae* bacteremias. Mortality at 30 days occurred in 12.3% and 3.7% in the piperacillin/tazobactam and meropenem groups, respectively, which failed to demonstrate the noninferiority of piperacillin/tazobactam. 

In our study, most patients had empirically received cefepime. For definitive therapy in the ESBL group, the majority received a carbapenem, but 8 (16%) did not. The most common noncarbapenem agent used for definitive therapy in the ESBL group was cefepime (5/8 (62.5%)), likely representing a continuation of empirical antibiotics. Three of the five patients (60%) who had received cefepime for definitive therapy died. Some of these patients on cefepime had died before culture results were available, which likely accounts for why a carbapenem was not used. The treatment of ESBL infections with cefepime based on in vitro sensitivity is controversial. No prospective clinical trials have evaluated it for this indication, but observational studies showed conflicting results [26,27,28,29]. Wang and colleagues compared empiric cefepime and carbapenems for patients with ESBL bacteremia, and found that 14-day mortality was higher in the cefepime group (41% vs. 20%) [26]. Additionally, cefepime trended towards a higher risk of mortality in the propensity-matched cohort (hazard ratio (HR), 2.87; 95% CI, 0.88–2.66). Lee and colleagues, who compared cefepime to carbapenems, had a similar outcome, demonstrating higher rates of clinical and microbiological failure, and 30-day mortality in patients who had received cefepime for definitive therapy [27]. On the other hand, Vu and colleagues performed a retrospective study in 114 patients and compared carbapenems (*n* = 74) to noncarbapenems, including cefepime (*n* = 30) and piperacillin/tazobactam (*n* = 10) [28]. In-hospital mortality was not significantly different between the carbapenem and noncarbapenem groups (8% vs. 3%, *p* = 0.42). Frescas and colleagues also found no difference in 14-day (5.6% vs. 2.8%, *p* = 1.00) or 30-day (11.1% vs. 2.8%, *p* = 0.255) mortality between patients who had received cefepime or meropenem for ceftriaxone resistance, cefepime-susceptible *E. coli*, and *K. pneumoniae* [29]. These four studies had several limitations: the retrospective designs and small patient populations; therefore, additional data are needed to determine if cefepime should be used for serious ESBL infections. The mortality rate in patients who had received cefepime for definitive therapy for ESBL-E bacteremia was high in our population, so cefepime should be used with caution for these infections. This aligns with the IDSA antimicrobial-resistance guidance suggesting against using cefepime for nonurinary infections [1]. 

Antibiotic selection remains an important component of optimizing clinical outcomes for the treatment of ESBL infections. Both antibiotic-stewardship (AS) and non-AS pharmacists could assist in the initiation of an appropriate therapy. For example, one institution trained all pharmacists to review the rapid diagnostic results of blood cultures, which included the identification of ESBL production (CTX-M only) [30]. This intervention demonstrated a significant reduction in the time to change to the targeted antimicrobial agent, and decreased the length of stay in intensive care units. Pharmacy trainees could also participate in carrying out similar clinical interventions under the supervision of preceptors [31,32]. There are limited data evaluating the impact of pharmacy trainee review of microbiological cultures results and antibiotic selection [31]; however, studies demonstrated positive results with pharmacy student involvement in other clinical pharmacy services, such as anticoagulation [32]. Future studies may be able to quantify the impact of pharmacy trainee interventions on management of patients with drug-resistant infections. 

When evaluating the management of patients with ESBL or non-ESBL infections, we identified opportunities for improvement on the basis of recently published studies, including the duration of antimicrobial therapy and the transition to an oral therapy. The duration of antibiotic therapy in the ESBL and non-ESBL groups was about 2 weeks. While some patients may have required extended therapy, this duration was likely unnecessarily long, since most patients had bacteremia due to a UTI source. Heil and colleagues suggested defining uncomplicated Gram-negative bacteremia on the basis of the source of infection (UTI, intra-abdominal or biliary infections, catheter-related bacteremia, uncomplicated pneumonia, and skin and soft tissue infections), source control, lack of immunocompromise, and rapid clinical improvement [33]. Many of our patients would be categorized as uncomplicated on the basis of this definition. In patients with uncomplicated Gram-negative bacteremia, 7 days are noninferior to 14 days of therapy in terms of all-cause mortality, complications, and readmission or extended hospitalization [34]. Prolonged therapy could increase the risk of antibiotic-related adverse effects and antibiotic resistance, and lead to longer lengths of stay [35]. For this reason, it is important to limit antimicrobial courses when possible. Though there are limited data on optimal treatment duration for ESBL bacteremias, one retrospective study compared short (≤10 days) and long (>10 days) courses of antibiotics for patients with ESBL *E. coli* bacteremias [36]. A total of 856 patients were included (426 in the short-course and 430 in the long-course group). The source of infection for most patients was the urinary tract (51%). Median duration was 8 days (IQR 6–9) in the short-course group, and 15 days (IQR 12–20) in the long-course group. There was no difference in 90-day mortality (4.9% vs. 6%, *p* = 0.55), suggesting that this duration could likely be applied on the basis of uncomplicated versus complicated Gram-negative bacteremia to patients with ESBL infections. 

Significantly more patients in the non-ESBL group were transitioned to oral therapy. While there are fewer oral options to treat ESBL-producing organisms, and there is a higher rate of coresistance in ESBL-expressing isolates, almost 40% of the patients’ cultures in the ESBL group showed susceptibility to trimethoprim–sulfamethoxazole or a fluoroquinolone. The lack of oral therapy in this group may reflect the pervasive belief that parenteral is superior to oral antibiotic therapy, which was recently challenged [37]. Tamma and colleagues conducted a retrospective study comparing 30-day mortality in patients who had undergone oral stepdown versus those who had continued parenteral therapy for Enterobacteriaceae bacteremia. A matched cohort of 1478 patients (739 in each group) found no difference in mortality (13.1% vs. 13.4%; HR 1.03; 95% CI 0.82–1.30). The analysis of mortality in patients who had received high- versus low-bioavailability antibiotic agents also found no difference (11% vs. 12.3%; HR 1.05, 95% CI 0.67–1.66). However, highly bioavailable options are generally preferred, and most antibiotics with lower bioavailability, such as many oral beta-lactams, cannot be used for ESBL infections due to resistance. More education is likely needed regarding the benefits of transitioning to oral therapy. In addition, pharmacists are often involved in converting medications from parenteral into oral therapy in hospitalized patients, and may be able to increase the use of oral agents for ESBL-E bacteremias. A recent study evaluated the impact of a pharmacist-driven intervention to improve oral antibiotic selection and duration for common infections at hospital discharge [38]. Results demonstrated an increase in guideline-concordant prescriptions from 36% to 81.5% (*p* < 0.001) following the intervention. 

These findings highlight the consequences of antimicrobial use. Antimicrobials are critical medications for which great care should be taken to preserve their activity. In this study, patients that had received more than one antibiotic course in the past 90 days were over three times more likely to have an ESBL infection than those who had not. While many of these regimens were likely necessary, as much as 50% of antimicrobial use in the United States may be inappropriate [39,40]. Antimicrobial stewardship efforts to decrease antibiotic use and durations could play a role in slowing the rising incidence of ESBL-E infections [41]. How to best incorporate the identified risk factors into clinical practice is unclear. A history of ESBL infections is a widely accepted predictor of ESBL infections [18,19]. In patients with a history of ESBL infections, empirical carbapenem therapy is reasonable. All patients included in this study who had had a history of an ESBL infection had a current ESBL infection. Incorporating the risk factor of recent antibiotic use into clinical practice is challenging. While these patients were at an increased risk of ESBL infections, the indiscriminate use of carbapenems could select for carbapenem resistance. Practitioners must balance the benefit of potentially starting patients earlier on more appropriate therapy with the risks of resistance. These decisions are likely made case by case, considering patient-specific characteristics and the risk of delayed appropriate therapy. One way this could be accomplished is with an ESBL prediction score, such as the one described by Augustine and colleagues [18]. Pharmacists and other healthcare providers could incorporate a similar algorithm to the one proposed in that article, which assists in selecting carbapenems or noncarbapenems on the basis of the risk of ESBL-E bacteremia, the severity of the illness, and Pitt bacteremia score. 

This study has several limitations. Due to the retrospective nature, information must have been clearly documented for assessment to be accurate. In addition, this was a single-center study, and the sample was small, which may have limited the ability to identify certain less frequent risk factors including recent urologic procedure. All-cause rather than infection-related mortality was evaluated. This could have affected the mortality, accounting for the lack of difference between the ESBL and non-ESBL groups, which had been described in previous studies. In addition, mortality in our study was lower than what had been reported, which may have been due to infection characteristics such as the sources of infection. The appropriateness of therapy was also not assessed, which may have impacted the clinical outcomes. 

## 5. Conclusions

This study identified that previous ESBL infections and the receipt of more than one antibiotic in the past 90 days represent risk factors for the development of ESBL-E infections. Though this study has limitations, such as the smaller population, which reinforces antimicrobial exposure as an important factor in the acquisition of antimicrobial-resistant infections. Knowledge of the risk factors could aid pharmacists and pharmacy trainees in assisting with optimal antibiotic selection, ensuring that patients at a high risk for ESBL infections receive early and effective therapy while minimizing the overuse of carbapenems. 

## Figures and Tables

**Table 1:** Baseline demographics.

Variable *n* (%) or Median (IQR)	Non-ESBL (*n* = 100)	ESBL (*n* = 50)	*p*-Value
Age	61.5 [52–72]	58 [49–68]	0.190
Female	46 (46)	22 (44)	0.817
African American	70 (70)	29 (58)	0.144
Organisms			1.000
*Escherichia coli*	76 (76)	38 (76)	
*Klebsiella pneumoniae*	24 (24)	12 (24)	
Penicillin or Cephalosporin Allergy	16 (16)	8 (16)	1.000
Infection source			
Urinary tract	58 (58)	27 (54)	0.641
Intra-abdominal	17 (17)	2 (4)	0.024
Lower respiratory tract	9 (9)	7 (14)	0.350
Bone/joints	1 (1)	3 (6)	0.108
Skin/soft tissue	0	2 (4)	0.110
Other	3 (3)	4 (8)	0.096
Unknown	12 (12)	5 (10)	0.716

IQR: interquartile range; ESBL: extended-spectrum beta-lactamases.

**Table 2:** Antibiotic regimen characteristics.

Variable *n* (%) or Median [IQR]	Non-ESBL (*n* = 100)	ESBL (*n* = 50)	*p*-Value
Empirical antibiotic agent			
Cefepime	49 (49)	24 (48)	0.908
Piperacillin-tazobactam	37 (37)	12 (24)	0.110
Meropenem	2 (2)	11 (22)	<0.001
Ceftriaxone	10 (10)	2 (4)	0.339
Other	2 (2)	1 (2)	1.000
Days to definitive antibiotic initiation	4 (2–6)	5.5 (3.75–9)	0.014
Total duration	13 (8–16)	14 (8–18)	0.157
IV duration	6 (4–11)	14 (7.75–18)	<0.001
Oral stepdown	50 (50)	2 (4)	<0.001
Definitive treatment with a carbapenem	3 (3)	42 (84)	<0.001

IQR: interquartile range; ESBL: extended-spectrum beta-lactamases; IV: intravenous.

**Table 3:** Univariate analysis of risk factors.

Variable *n* (%) or Median (IQR)	Non-ESBL (*n* = 100)	ESBL (*n* = 50)	*p*-Value
Previous ESBL infection	0 (0)	9 (18)	<0.001
Quick Pitt bacteremia score	1 [0–2]	1 [0–3]	0.518
Charlson comorbidity index	5 [3–7]	4 [3–6]	0.817
Infection type			<0.001
Community-acquired	39 (39)	5 (10)	
Healthcare-associated	40 (40)	24 (48)	
Nosocomial-acquired	21 (21)	21 (42)	
Antibiotic courses in past 90 days	2 [1–2.5]	3 [2–6]	<0.001
Admitted to ICU during hospitalization	42 (42)	25 (50)	0.353
Urinary catheter	19 (19)	16 (32)	0.076
Central line > 2 days	11 (11)	13 (26)	0.018
Kidney stones	13 (13)	3 (6)	0.190
End-stage renal disease	4 (4)	5 (10)	0.161
Long-term care facility	8 (8)	9 (18)	0.069
Previous admission in the past 12 months	1 [0–2]	1 [0–2.25]	0.107
Urologic procedure	1 (1)	1 (2)	1.000
Immunosuppression			0.04
None	71 (71)	25 (50)	
One agent	13 (13)	12 (24)	
Corticosteroids	5 (5)	2 (4)	
Chemotherapy	3 (3)	5 (10)	
Radiation therapy	1 (1)	0 (0)	
Immunomodulating agent	4 (4)	5 (10)	
Multiple agents	16 (16)	13 (26)	

IQR: interquartile range; ESBL: extended-spectrum beta-lactamases; ICU: intensive care unit.

**Table 4:** Multivariate analysis of risk factors.

Final Model Variable	Odds Ratio	95% Confidence Interval	*p*-Value
Central line > 2 days	1.625	0.547–4.823	0.382
Community infection	0.412	0.125–1.363	0.146
Immunosuppression	1.783	0.793–4.010	0.162
>1 previous antibiotic	3.448	1.494–7.957	0.004
Long-term care facility	2.889	0.916–9.110	0.070

## Data Availability

Not applicable.
